# Identification of a cold-tolerant locus in rice (*Oryza sativa* L.) using bulked segregant analysis with a next-generation sequencing strategy

**DOI:** 10.1186/s12284-018-0218-1

**Published:** 2018-04-18

**Authors:** Jian Sun, Luomiao Yang, Jingguo Wang, Hualong Liu, Hongliang Zheng, Dongwei Xie, Minghui Zhang, Mingfang Feng, Yan Jia, Hongwei Zhao, Detang Zou

**Affiliations:** 1 College of Agriculture, Northeast Agricultural University, Harbin, 150030 China; 2 The Institute of Industrial Crops of Heilongjiang Academy of Agricultural Sciences, Harbin, 150086 China; 3 College of Life Science, Northeast Agricultural University, Harbin, 150030 China

**Keywords:** *Oryza sativa* L, Cold tolerance, QTL, Seq-BSA, Candidate gene

## Abstract

**Background:**

Cold stress can cause serious abiotic damage that limits the growth, development and yield of rice. Cold tolerance during the booting stage of rice is a key factor that can guarantee a high and stable yield under cold stress. The cold tolerance of rice is controlled by quantitative trait loci (QTLs). Based on the complex genetic basis of cold tolerance in rice, additional efforts are needed to detect reliable QTLs and identify candidate genes. In this study, recombinant inbred lines (RILs) derived from a cross between a cold sensitive variety, Dongnong422, and strongly cold-tolerant variety, Kongyu131, were used to screen for cold-tolerant loci at the booting stage of rice.

**Results:**

A novel major QTL, *qPSST6*, controlling the percent seed set under cold water treatment (PSST) under the field conditions of 17 °C cold water irrigation was located on the 28.4 cM interval on chromosome 6. Using the combination of bulked-segregant analysis (BSA) and next-generation sequencing (NGS) technology (Seq-BSA), a 1.81 Mb region that contains 269 predicted genes on chromosome 6 was identified as the candidate region of *qPSST6*. Two genes, *LOC_Os06g39740* and *LOC_Os06g39750*, were annotated as “response to cold” by gene ontology (GO) analysis. qRT-PCR analysis revealed that *LOC_Os06g39750* was strongly induced by cold stress. Haplotype analysis also demonstrate a key role of *LOC*_*Os06g39750* in regulating the PSST of rice, suggesting that it was the candidate gene of *qPSST6*.

**Conclusions:**

The information obtained in this study is useful for gene cloning of *qPSST6* and for breeding cold-tolerant varieties of rice using marker assisted selection (MAS).

**Electronic supplementary material:**

The online version of this article (10.1186/s12284-018-0218-1) contains supplementary material, which is available to authorized users.

## Background

Rice, a staple food crop cultivated worldwide, feeds more than half of the world’s population. Rice originates from tropical and subtropical regions and is more sensitive to low temperature than other crops originating in temperate zones (Wang et al. 2016). In high-latitude or high-altitude regions of Asia, Europe, America and other rice cultivation areas of the world, the temperature is not consistently high enough for the growth of rice. Remarkably, low temperatures that occur frequently during the reproductive stage of rice can cause a fatal yield loss (Jena et al. 2012; Shirasawa et al. 2012). Male sterility, arising from low temperature (lower than 19 °C) during the period of microspore development at the booting stage, is the key reason for the reduction of percent seed set and the resulting loss in yield (Satake and Hayase 1970). Therefore, the breeding of cold-tolerant varieties at the booting stage is an effective method to maintain high and stable yields in rice cultivation regions.

The cold tolerance of rice is a complex trait, and many methods have been established to evaluate and select cold-tolerant varieties of rice (Zhang et al. 2014). A cold-water irrigation system has been developed as a reliable identified method of determining cold tolerance at the booting stage of rice. Rice plants are maintained in a cold deep-water irrigated pool during the entire booting stage, and the spikelet fertility was used to examine the cold tolerance of rice varieties (Shirasawa et al. 2012). This method exposes rice plants in field growth conditions, and its accurate evaluation results are still widely used for selecting cold-tolerant lines and developing cold-tolerant rice varieties (Matsunaga 2005; Jia et al. 2015).

Many studies agree that the cold tolerance of rice is controlled by quantitative trait loci (QTLs) (Andaya and Mackill 2003a; Zhang et al. 2005; Suh et al. 2010; Zhang et al. 2014). QTL mapping is the main approach to excavate and clone cold-tolerant related genes in rice. At the booting stage of rice, a number of QTLs about cold tolerance have been reported, including *qCTB3* (Andaya and Mackill 2003b), *qPSST-3* (Suh et al. 2010) and *qLTB3* (Shirasawa et al. 2012) on chromosome 3, *Ctb1* (Saito et al. 2004), *Ctb2* (Saito et al. 2001), *qCTB-4-1*, *qCTB-4-2* (Xu et al. 2008), and *CTB4a* (Zhang et al. 2017) on chromosome 4, and *qPSST-7* (Suh et al. 2010) and *qCTB7* (Zhou et al. 2010) on chromosome 7. For the different rice materials and research backgrounds, QTLs for cold tolerance at the booting stage were mapped at the various locations on different chromosomes. However, the above QTLs identified by the biparental cross linkage mapping method are labor and time intensive to map the genotypes a large number of individuals in the segregated population and to finely map the target QTL (Salvi and Tuberosa 2005).

A bulked segregant analysis (BSA) is a simple and rapid approach to identify molecular markers tightly linked to the target genes or QTLs (Michelmore et al. 1991). With the rapid development of next-generation sequencing (NGS) technology, the combination of the BSA with NGS strategy (Seq-BSA) is becoming a widely used approach in the mapping of major QTLs and gene identification (Wenger et al. 2010; Takagi et al. 2013). This strategy has been demonstrated in many plants, such as Arabidopsis (Schneeberger et al. 2009), rice (Abe et al. 2012; Wambugu et al. 2017), soybean (Song et al. 2017), wheat (Trick et al. 2012), pigeon pea (Singh et al. 2016), sunflower (Livaja et al. 2013) and sorghum (Han et al. 2015), and it has identified some QTLs and genes for important traits. In the present study, two strategies, traditional QTL mapping and Seq-BSA were employed to identify the genes for cold tolerance at the booting stage in recombinant inbred lines (RILs) derived from a cross between a cold sensitive variety, Dongnong422, and a strongly cold-tolerant variety, Kongyu131. The results contribute to the understanding of the genetic bases for cold tolerance at the booting stage, and future cloning of the candidate gene will facilitate the molecular breeding of cold tolerance in rice.

## Methods

### Plant materials

Two *japonica* varieties, “Dongnong422” (DN422) and “Kongyu131 (KY131)” were used as parental lines to develop the RIL population. DN422 is a cold-sensitive variety that was obtained from Northeast Agriculture University. KY131, a strongly cold-tolerant variety, is widely cultivated in the northeast region of China. The mapping populations of 190 F_7_, F_8_ and F_9_ RILs were produced by single seed descent (SSD) from an F_2_ population of a cross between DN422 and KY131.

### Cold tolerance evaluation

An evaluation of cold tolerance was performed on the experimental farm of Northeast Agricultural University, Harbin, China (47°98′N, 128°08′E) in 2014, 2015 and 2016. The parents and RIL populations were grown in a randomized block design with two replications of double row plots, a 2-m row length, a 30-cm row spacing and a 10-cm hill spacing. The 190 RILs were divided into three groups, early maturing, middle-maturing and late-maturing groups, according to their heading dates. DN422 belongs to the late-maturing group, and KY131 belongs to the early maturing group. For the evaluation of cold tolerance, each group was irrigated with 17 °C water in an irrigated pool (25 m × 5 m) independently from the panicle initiation stage (approximately 35 days after transplanting for the early maturing group, approximately 40 days after transplanting for the middle-maturing group, and approximately 45 days after transplanting for the late-maturing group) to the full heading stage. When the auricle of the flag leaf is approximately 5 cm below the auricle of the penultimate leaf on each plant, the pollen should have undergone meiosis, which was indicative of panicle initiation stage (Satake and Hayase 1970). Water at 17 °C was prepared in a storage pool with a cool and warm water mixture measured by the temperature sensors. Flood irrigation was performed from the inlet to the outlet (5 m) of the irrigated pool. The depth of the irrigated water was 18–20 cm, and the irrigated time was from 8 a.m. to 16 p.m. every day.

The percent seed set under the cold-water treatment (PSST), the ratio of the number of fertile seeds in the number of total seeds, was used as the index of cold tolerance. A basic statistical analysis was implemented by the SPSS16.0 software (SPSS Inc., Chicago, IL, USA). The mean data of PSST in RILs over the two replications was used for QTL analysis, and the extreme cold-tolerant and sensitive plants in the RIL population used for Seq-BSA were selected according to the mean data of F_7_, F_8_ and F_9_ RILs.

### QTL analysis

One hundred eighty-five polymorphic SSR markers between DN422 and KY131 covering the rice genome were used for genotyping the RIL population. A PCR was performed according to the procedure of Chen et al. (1997), and the PCR products were then separated on a 6% polyacrylamide gel, followed by silver staining.

The MAP function of QTL IciMapping 3.2 (Wang et al. 2012) was used to construct the genetic linkage map of the RIL population, and the Kosambi’s mapping function was used to calculate the genetic distances. QTLs were detected using the inclusive composite interval mapping (ICIM) module of QTL IciMapping 3.2. The threshold of the LOD score for declaring the presence of a significant QTL was determined by the permutation test with 1000 repetitions at *P* < 0.05. The QTL was named according to the trait and its chromosome location.

### Construction of segregating pools and sequencing

For Seq-BSA, two DNA pools were developed by selecting the extreme cold-tolerant and extreme cold-sensitive individuals according to the PSST of the RIL population in the range from 0.50–0.95. The tolerant pool (T-pool) was made by mixing equal amounts of DNA from 20 extreme cold-tolerant RILs with a PSST above 0.90, and the sensitive pool (S-pool) was made by mixing equal amounts of DNA from 20 extreme cold-sensitive RILs with a PSST below 0.63 (Additional file 2: Table S1). The DNA isolated from DN422 and KY131 and the two DNA pools were prepared for sequencing.

Libraries for all the DNA pools were prepared according to the Illumina TruSeq DNA sample Preparation v2 Guide. The DNA libraries were sequenced on Illumina MiSeq platform using MiSeq Reagent Kit v2 (500 cycles) (Illumina Inc., San Diego, CA, USA). The short reads from both parents and the two DNA pools were aligned to *Nipponbare* reference genome (IRGSP 2005) using the BWA software (Li and Durbin 2009). Reads of the T-pool and S-pool were separately aligned to KY131 and DN422 consensus sequence reads to call SNPs with the SAM tools software (Li and Durbin 2009).

### Analysis of the Seq-BSA data

According to the locating results of clean reads among the reference genome, duplicate reads were removed using the Picard tool (http://sourceforge.net/projects/picard/), and GATK software (McKenna et al. 2010) was used to perform the local realignment and base recalibration to insure the accuracy of the SNP detecting. The SNP loci between the test samples and reference genome were obtained using the GATK software according to the best practices on the GATK website (https://www.broadinstitute.org/gatk/guide/best-practices.php). All the SNP loci between the test samples were summarized according to the alignment results of test samples and the reference genome.

The Euclidean distance (ED) and SNP-index were calculated to identify the candidate regions of the genome associated with PSST. The ED algorithm is a method of searching markers with significant differences between the pools according to the sequencing data and evaluating the associated regions between markers and traits (Hill et al. 2013).

The calculation formula of the ED algorithm was as follows:

$$ ED=\sqrt{{\left( Aaa- Aab\right)}^2+{\left( Caa- Cab\right)}^2+{\left( Gaa- Gab\right)}^2+{\left( Taa- Tab\right)}^2} $$

where A_aa_, C_aa_, G_aa_ and T_aa_ represent the frequency of bases A, C, G and T in the T-pool, respectively. A_ab_, C_ab_, G_ab_ and T_ab_ represent the frequency of bases A, C, G and T in the S-pool, respectively. The depth of each base in different pools and the ED value of each SNP loci were calculated. To eliminate the background noise, the ED value was powered, and the ED^5^ was used as the associated value (Hill et al. 2013).

The SNP-index association analysis is a method used to calculate genotype frequency differences between two pools (Takagi et al. 2013; Fekih et al. 2013). A SNP-index is the proportion of reads harboring the SNP that are different from the reference sequence. The Δ(SNP-index) of each locus was calculated by subtraction of the SNP-index of the T-pool from that of the S-pool. SNP-index = 0 if the entire short reads contain genomic fragments from DN422; SNP-index = 1 if all the short reads were from KY131. The average of the SNP-index was calculated in a 1 Mb interval using a sliding window analysis with 10 kb. The SNP-index graphs of the T-pool, S-pool and corresponding Δ(SNP-index) graphs were plotted. The statistical confidence intervals of Δ(SNP-index) were calculated under the null hypothesis of no QTLs following the description of Takagi et al. (2013).

### qRT-PCR analysis of the candidate genes

Two parents, DN422 and KY131, were used to identify the expression patterns of the putative candidate genes. Samples were collected when they reached the panicle initiation stage after cold water irrigation in 2017. Plants with normal temperature water irrigation were used as the control. The leaves of the two parents were collected at 0, 1, 3, 5, 7, 9, 11 and 13 d, for three repetitions. The samples were placed in liquid nitrogen immediately and then stored at − 80 °C for total RNA isolation.

Total RNA was isolated using the Trizol reagent (Invitrogen) according to the manufacturer’s instructions and was purified using the DNA-free RNA kit. The first-strand cDNA was synthesized using the Fermentas RevertAid First Strand cDNA Synthesis Kit. A pair of primers was designed for each candidate gene using the Primer Premier 5.0 software. The housekeeping gene *Actin1* (*Os05g36290*) was used as the internal control (Siahpoosh et al. 2011). The information of the specific primers is provided in Additional file 2: Table S2. The qRT-PCR reactions were performed using a Roche LightCycler 2.10 with a 2 × SYBR Green I PCR Master Mix. The PCR reaction procedure was as follows: 95 °C for 5 min; 45 cycles of 95 °C for 10 s, 60 °C for 20 s, and 72 °C for 20 s. The mean Ct values of all the biological replicates were normalized with the Ct values of *Actin1*. The relative expression level was calculated using the 2^-ΔΔCt^ method (Livak and Schmittgen 2001), where ΔΔCt = (Ct, _target_ - Ct, _actin_)_time *x*_ - (Ct, _target_ - Ct, _actin_)_time 0_.

### Sequencing of the nsSNP loci in RILs

Genomic DNA of the 190 RILs was extracted using the CTAB method (Murray and Thompson 1980).The eight SNP loci in *LOC*_*Os06g39750* were sequenced to identify the haplotypes of RIL population. Primers for amplifying the SNP were designed using the Primer Premier 5.0 software, and the sequences of specific primers are provided in Additional file 2: Table S3.

Total 20 μL PCR reaction mixture contained 2 μL of genomic DNA (50 ng/μL), 1.5 μL of forward primer (10 μM), 1.5 μL of reverse primer (10 μM), 5 μL ddH_2_O, and 10 μL of Pfu Master Mix (CWBio, Inc., Beijing, China) including Taq DNA Polymerase, PCR Buffer, Mg^2+^ and dNTPs. The PCR reaction was carried out in Eppendorf Mastercycler 5333 thermal cycler, and the procedure was same as the above qRT-PCR. The amplified products were checked by electrophoresis in 1% agarose gel. PCR products were directly sequenced by the BGI Life Tech Co., Ltd.

## Results

### Phenotypic characterization of the cold tolerance in DN422/KY131 RILs

**Fig. 1:**
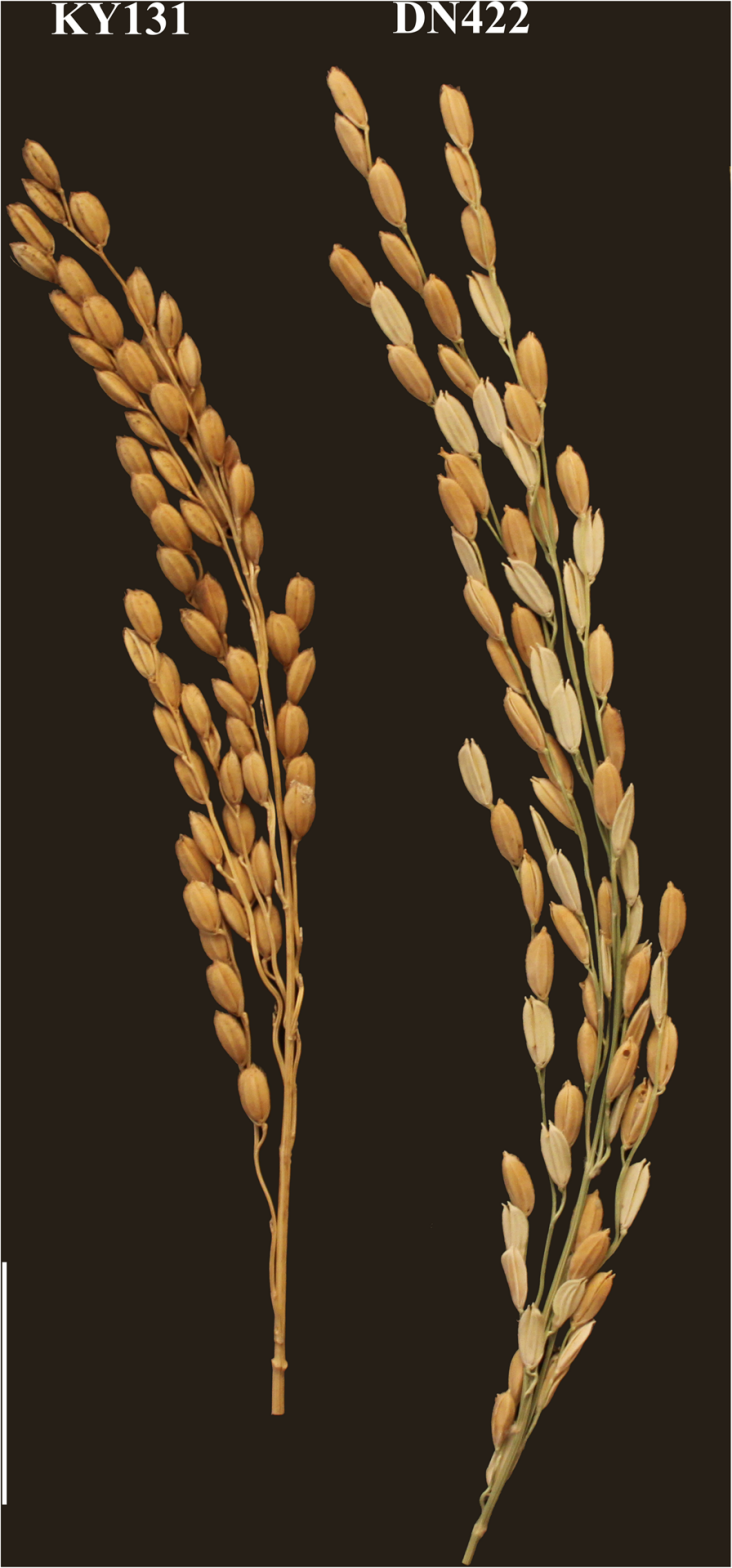
Panicles of DN422 and KY131 planted under cold water irrigation condition. Scale bars, 3 cm

In the present study, two *japonica* varieties, DN422 and KY131, were crossed to develop the RIL populations for the QTL analysis and the Seq-BSA of cold tolerance. The phenotypic data were collected using the F_7_, F_8_ and F_9_ RILs in 2014, 2015 and 2016, continuously, and the basic statistical analysis of the tested materials is provided in Table 1. Extensive phenotyping data under cold stress and control conditions for heading time, plant height, panicle number, and grain yield per plant were provide in Additional file 2: Table S4 to insights into the cold tolerance of the test materials. The mean PSST values of DN422, KY131 and the RIL population in the 3 years were 0.59, 0.92 and 0.76, respectively, and their mean percent seed set values under normal water irrigation (PSSN) were 0.90, 0.97 and 0.89, respectively. The correlation analysis showed a significantly positive correlation among the PSST in 2014, 2015 and 2016, respectively, and the PSSN had the same pattern (Additional file 2: Table S5). This indicates that the cold-water irrigation was moderate and could differentiate the plants between cold stress and control effectively. The PSST values of the cold-tolerant parent KY131 were extremely significant higher than those of the cold-sensitive DN422 in all 3 years (Fig. 1, Table 1), indicating the stronger cold tolerance of KY131 compared to DN422. Among the RIL populations, the absolute values of skewness and kurtosis for PSST in 3 years were all close to 1 which indicate that the data of PSST were suitable for the QTL analysis (Table 1) (Li et al. 2012).

**Table 1:** PSST and PSSN of parents and RIL population in 3 years

Traits	Year	DN422^a^	KY131	RIL populations^b^				
				Mean	Range	SD	Skewness	Kurtosis
PSST	2014	0.57 ± 0.09	0.92** ± 0.11	0.78	0.42~ 0.94	0.14	− 1.08	1.16
	2015	0.61 ± 0.13	0.93** ± 0.08	0.79	0.50~ 0.98	0.12	−0.42	− 0.87
	2016	0.58 ± 0.07	0.91** ± 0.14	0.70	0.31~ 0.99	0.15	0.02	−0.65
	Mean	0.59 ± 0.10	0.92** ± 0.09	0.76	0.50~ 0.95	0.10	−0.10	−0.53
PSSN	2014	0.91 ± 0.05	0.97 ± 0.09	0.95	0.87~ 1.00	0.07	0.25	0.43
	2015	0.88 ± 0.06	0.98 ± 0.11	0.85	0.66~ 0.99	0.03	−0.27	−0.99
	2016	0.90 ± 0.14	0.96 ± 0.06	0.87	0.67~ 0.99	0.08	−0.36	−0.49
	Mean	0.90 ± 0.11	0.97 ± 0.08	0.89	0.73~ 0.99	0.06	−0.07	−0.93

^a^ Means ± SD (standard deviation)

^b^ Population sample size *n* = 190, replications *r* = 2

*,** significance at the level of 5 and 1%, respectively, according to Student’s t-test

### SSR-based QTL mapping

One thousand SSR markers with a uniform distribution throughout the 12 rice chromosomes were selected to screen the polymorphism between DN422 and KY131. Among them, 158 SSR markers were polymorphic between the two parents and were used to construct the genetic linkage map of the RIL population. The map included 12 chromosomes and covered 2355.3 cM of the rice genome with an average distance of 14.91 cM between markers.

In total, three QTLs for the PSST located on chromosomes 5, 6 and 7 were detected by using the ICIM module of QTL IciMapping 3.2 in 3 years (Table 2). The total phenotypic variation explained (PVE) for all identified QTLs ranged from 9.01 to 47.94%. *qPSST5* and *qPSST7* were detected in only 1 year, and their PVE were 9.01% and 14.95%, respectively. Their facticity in controlling the PSST of rice is suspicious. Remarkably, *qPSST6* in the RM20261-RM20356 interval of chromosome 6 was detected in all 3 years, and the PVE values of the 3 years were 24.30%, 47.94% and 19.11%, respectively, with a mean of 30.45%. In addition, their additive effect values were all negative, indicating that KY131 had a positive effect on increasing the PSST. Thus, *qPSST6* is a major QTL for a high PVE and existed stably in the 3 years, suggesting a key role in controlling the PSST of rice. In contrast, six QTLs with small PVE for the PSSN were detected on chromosomes 2, 4, 6, 7, 8, and major QTL was not found (Additional file 2: Table S6).

**Table 2:** QTLs for PSST of rice detected in 3 years

QTL	Years	Chr.^a^	Marker interval	LOD^b^	A^c^	PVE%^d^
*qPSST5*	2015	5	RM146-RM18332	4.69	0.04	9.01
*qPSST6*	2014	6	RM20261-RM20356	5.43	−0.05	24.30
	2015	6	RM20261-RM20356	12.29	−0.08	47.94
	2016	6	RM20261-RM20356	5.52	−0.07	19.11
*qPSST7*	2016	7	RM1335-RM182	3.88	0.02	14.95

^a^ Chromosome

^b^ Logarithm of odd score for each QTL

^c^ Additive effect of the QTL. A positive value indicates that DN422 contributes the positive allele; a negative value indicates that KY131 contributes the positive allele

^d^ Phenotypic variation explained by A effects

### Sequencing of the parents and extreme pools

Twenty RILs with extreme cold tolerance (PSST ranged from 0.90–0.95) and 20 RILs with extreme cold sensitivity (PSST ranged from 0.50–0.63) were selected to prepare the T-pool and S-pool (Additional file 2: Table S1). Illumina high-throughput sequencing generated 233.62 million raw reads, and 232.30 million clean reads (99.43%) were obtained after filtering. The mean value of Q30 was 91.92%, indicating that most of the bases were high quality. The sequencing depths were 38-fold in DN422, 42-fold in KY131, 31-fold in the T-pool, and 45-fold in the S-pool, which could guarantee the accuracy of the BSA analysis (Additional file 1: Figure S1, Additional file 2: Table S7). There were 336,632 SNPs between DN422 and KY131, which included 36,448 non-synonymous SNPs (nsSNP); there were 184,917 SNPs between the T-pool and the S-pool, which included 18,013 nsSNP. The raw sequencing data were deposited in NCBI (https://www.ncbi.nlm.nih.gov/), and the accession number were SRR6327815, SRR6327816, SRR6327817, SRR6327818 for KY131, DN422, T-pool and S-pool, respectively.

### ED and SNP-index analysis

**Fig. 2:**
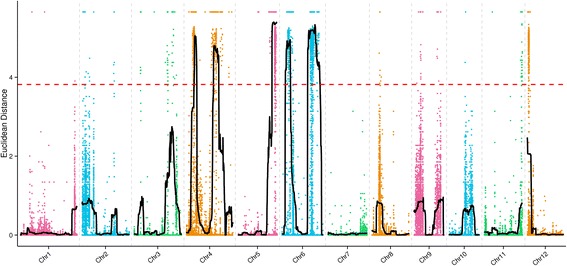
Identification of the hot-region for PSST through the ED association analysis method. X-axis represents the position of 12 chromosomes of rice and Y-axis represents the ED. The color dots represent the ED value of every SNP locus. The black lines show the ED value of fitting results, and the red imaginary lines show the association threshold of ED

The two analysis methods of Seq-BSA and the ED and SNP-index were used to identify the candidate genome regions associated with PSST. The 184,917 SNPs between the T-pool and the S-pool were used for an association analysis through the two methods. The association threshold of the ED method was 3.82, and six genome regions were significantly correlated with the PSST. The result of the ED association analysis is shown in Fig. 2 and Table 3. The candidate regions had a total size of 23.48 Mb, distributed on chromosomes 4, 5 and 6, and contained 4338 genes with 792 nsSNP loci among them.

**Table 3:** Information of the association region by the ED association analysis method

Chromosomes	Chromosome locations (bp)		Size (Mb)	Gene number
	Start	End		
4	5,830,000	7,910,000	2.08	324
4	20,560,000	24,640,000	4.08	770
5	26,010,000	29,950,000	3.94	873
6	2,530,000	6,440,000	3.91	756
6	19,480,000	28,910,000	9.43	1601
6	28,930,000	28,970,000	0.04	14
Total	–	–	23.48	4338

**Fig. 3:**
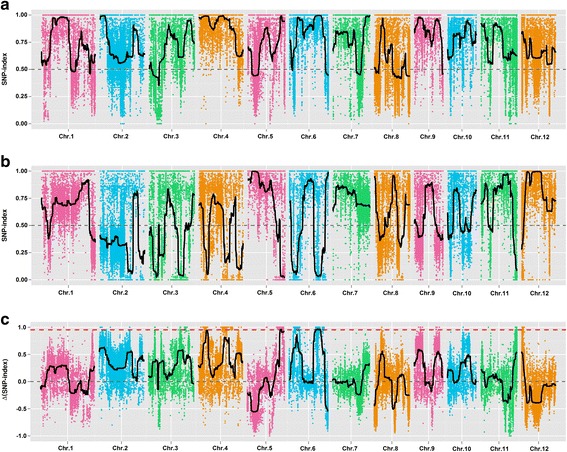
Identification of the hot-region for PSST through the SNP-index association analysis method. X-axis represents the position of 12 chromosomes of rice and Y-axis represents the SNP-index or Δ (SNP-index). The color dots represent the SNP-index or Δ (SNP-index) value of every SNP locus. The black lines show the SNP-index or Δ (SNP-index) value of fitting results. a: the SNP-index graph of T-pool. b: the SNP-index graph of S-pool. c: the Δ (SNP-index) graph. The red dotted line shows the association threshold value (0.9532)

The SNP-index of the T-pool and S-pool were calculated for each identified SNP in the genome, and an average SNP-index was computed in a 1 Mb interval using a 10-kb sliding window. By combining the information of the SNP-index of the T-pool and S-pool, the Δ(SNP-index) was calculated, and the Δ(SNP-index) trends were visualized by means of a sliding window. Using the association threshold of 0.9532, six genome regions distributed on chromosomes 5 and 6 that were significantly correlated with the PSST were identified. The total length of these regions was 3.68 Mb, and they contained 649 genes with 93 nsSNP loci (Fig. 3, Table 4). By comparing the association results of ED and the SNP-index methods, their intersections of the genome regions were consistent to the association regions of the SNP-index method (Table 4). The 3.68 Mb regions of chromosome 6 by Seq-BSA was the hot region for the PSST of rice.

**Table 4:** Information of the association region by the SNP-index association analysis method

Chromosomes	Chromosome locations (bp)		Size (Mb)	Gene number
	Start	End		
Chr5	26,700,000	27,370,000	0.67	158
Chr6	21,840,000	22,070,000	0.23	33
Chr6	22,140,000	24,450,000	2.31	388
Chr6	24,480,000	24,700,000	0.22	27
Chr6	24,750,000	24,830,000	0.08	16
Chr6	24,980,000	25,150,000	0.17	27
Total	–	–	3.68	649

**Fig. 4:**
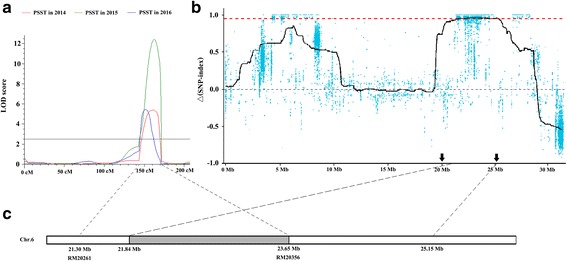
Mapping of *qPSST6* on chromosome 6 using the methods of QTL analysis and Seq-BSA. a: *qPSST6* detected in the RIL population by using the ICIM module of QTL IciMapping 3.2. The LOD scores are shown with three distinct peaks corresponding to *qPSST6* in 2014, 2015 and 2016, respectively. b: hot-region for PSST identified by SNP-index method on chromosome 6. The black line shows the Δ (SNP-index) value of fitting results. The red dotted line shows the association threshold value (0.9532), and the black arrows on the x-axis represent the location of hot-region for PSST. c: the intersection of *qPSST6* interval identified by QTL analysis and Seq-BSA. The gray region on the chromosome shows the 1.81 Mb intersection of *qPSST6*

To narrow the genome region of PSST further, mapping interval of the major QTL *qPSST6* was compared to the hot region of Seq-BSA. *qPSST6* was detected in the interval between RM20261-RM20356 with a genetic distance of 28.4 cM by using the ICIM module of QTL IciMapping 3.2. RM20261 is located at 21,304,727–21,304,750 bp, and RM20356 is located at 23,654,188–23,654,207 bp on chromosome 6 of the *Nipponbare* genome. *qPSST6* spanned a physical distance of 2.35 Mb and intersected with the hot region of Seq-BSA between 21,840,000 bp and 23,654,188 bp (Fig. 4). Thus, the region containing the PSST locus was narrowed to a region of 1.81 Mb, which contains 269 predicted genes.

### Identification of the candidate genes for PSST

To identify the candidate genes for PSST, the 269 predicted genes were searched in gene ontology (GO) (Ashburner et al. 2000), non-redundant protein (NR) (Deng et al. 2006), Swiss-Prot (Apweiler et al. 2004), Kyoto Encyclopedia of Genes and Genomes (KEGG) (Kanehisa et al. 2004) and Cluster of Orthologous Groups of proteins (COG) (Tatusov et al. 2000) databases by the BLAST software (Altschul et al. 1997). The results revealed that among the 269 predicted genes, 212 were successfully annotated. The most enriched terms of biological process ontology were metabolic processes, such as carbohydrate metabolic processes (GO: 0005975), DNA metabolic processes (GO:0006259), cellular macromolecule metabolic processes (GO:0044260), and nucleic acid metabolic processes (GO:0090304). The most enriched terms of molecular function were binding, such as nucleic acid binding (GO:0003676), iron ion binding (GO:0005506), and zinc ion binding, (GO:0008270). The most enriched terms of cellular component were membranes, such as chloroplast outer membranes (GO:0009707), cytoplasmic membrane-bounded vesicles (GO:0016023), and plasma membranes (GO:0005886). All the annotated information is listed in Additional file 2: Table S8. Out of the 212 annotated genes, only two, *LOC_Os06g39740* and *LOC_Os06g39750*, were annotated as the function of “response to cold (GO:0009409)”, suggesting their key roles in regulating cold tolerance in rice. Moreover, 13 predicted genes (4.83%) contained 52 nsSNP loci including three nsSNP in *LOC_Os06g39750*, but no non-synonymous substitution occurred in *LOC_Os06g39740* (Additional file 2: Table S9).

**Fig. 5:**
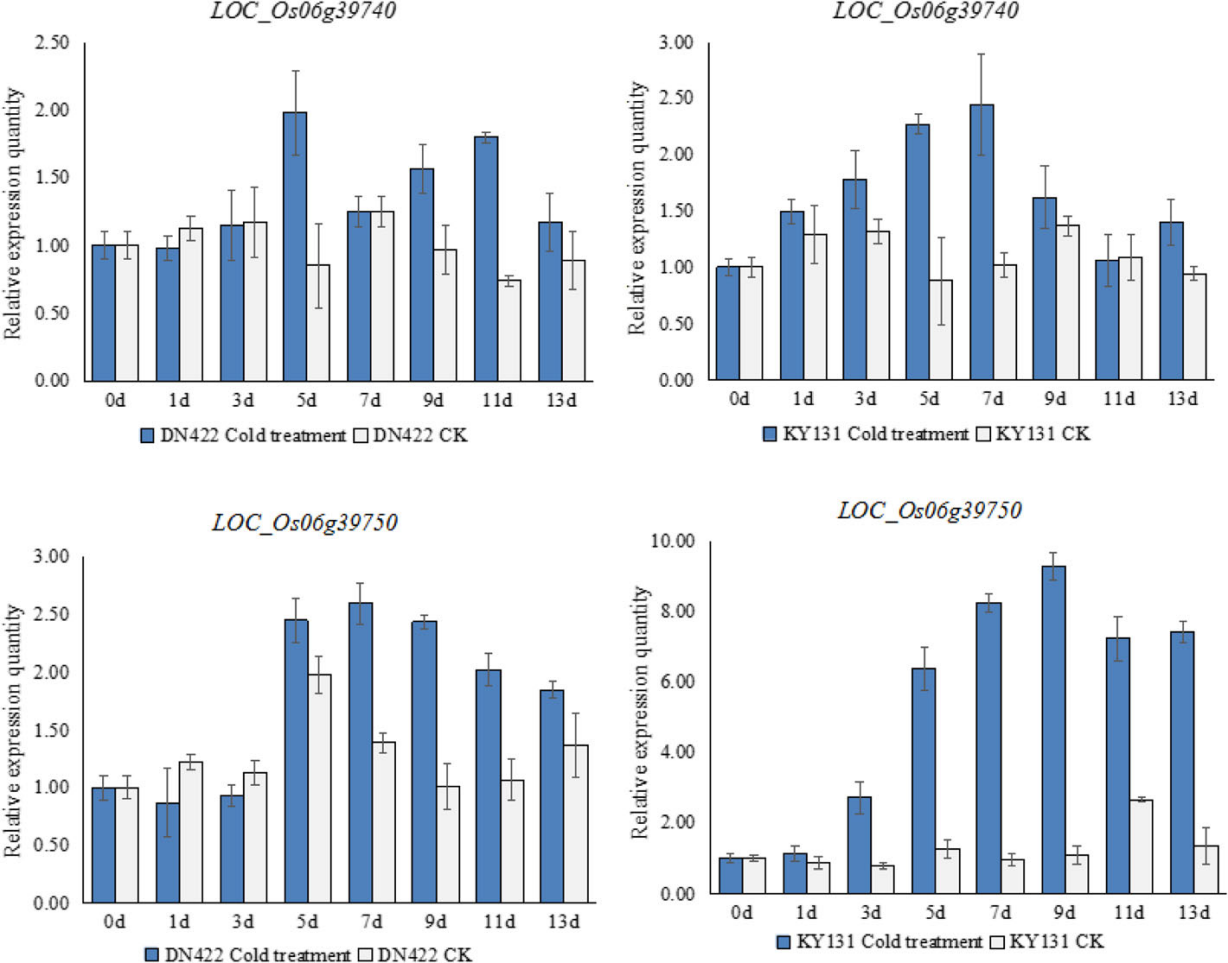
Expression patterns of *LOC_Os06g39740* and *LOC_Os06g39750* under the conditions of cold water irrigation and CK. Each line with different patterns on the X-axes from left to right indicate the sampling times of 0, 1, 3, 5, 7, 9, 11 and 13 day after cold water irrigating in KY131 and DN422. The Y-axes indicate the relative expression quantity. The error bars indicate SD for the data obtained in three biological replicates

To determine the expression patterns of the two genes annotated as “response to cold” in response to cold stress, a qRT-PCR was performed using the total RNA of the two parents DN422 and KY131 subjected to cold water conditions. Two primers pairs for the qRT-PCR analysis were designed based on the CDS sequences of the two genes (Additional file 2: Table S2). According to the qRT-PCR results (Fig. 5), *LOC_Os06g39740* was slightly induced by the cold stress after 3 days in DN422 and after 1 day in KY131. The largest relative expression quantity was nearly 2-fold at day 5 in DN422 and 2.5-fold at day 7 in KY131. The expression patterns of *LOC_Os06g39740* have no obvious up or down-regulation for all days at the control conditions in DN422 and KY131. *LOC_Os06g39750* exhibits a strong inducement by cold stress at days 5, 7, 9, 11, and 13 after treatment in KY131, and the highest relative expression quantity was above 9-fold at day 9. Compared to KY131, the up-regulated expression of *LOC_Os06g39750* under cold stress in DN422 were weak, and the highest relative expression quantity was approximately 2.6-fold at day 7. *LOC_Os06g39750* exhibited no obvious regularity in the control condition in both DN422 and KY131. The result of the qRT-PCR suggested that *LOC_Os06g39750* was the candidate gene controlling the PSST in rice.

**Fig. 6:**
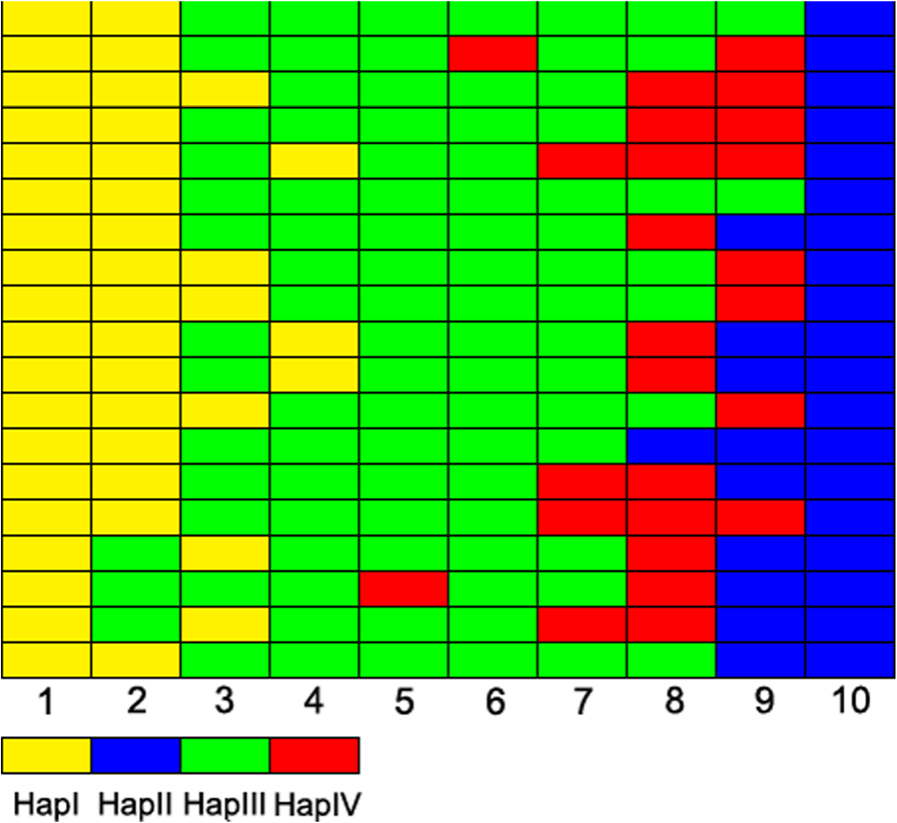
Haplotype distribution of *LOC*_*Os06g39750* in RIL population*.* Every block represents a RIL, and all RILs are arranged according to their PSST value. 1, 2, 3, 4, 5, 6, 7, 8, 9, 10 column represent the RILs with PSST of 0.56~ 0.63, 0.63~ 0.67, 0.68~ 0.70, 0.70~ 0.72, 0.73~ 0.75, 0.75~ 0.77, 0.77~ 0.82, 0.82~ 0.85, 0.85~ 0.90, 0.90~ 0.95 from top block to bottom block, respectively. Yellow, blue, green, and red block represent HapI, HapII, HapIII, and HapIV, respectively

Among the *LOC_Os06g39750*, eight SNP loci were identified, and they were all in the CDS region of *LOC_Os06g39750* (Additional file 2: Table S10). Sequencing of the eight SNP loci in *LOC*_*Os06g39750* revealed that four haplotypes (HapI, HapII, HapIII, and HapIV) existed among the RIL population (Fig. 6, Additional file 2: Table S11). HapI, DN422 genotype, which contains 20 RILs of S-pool and other 24 RILs. HapII, KY131 genotype, which contains 20 RILs of T-pool and other 9 RILs. HapIII contains 92 RILs, which has seven same SNPs to DN422, and only one same SNP to KY131. HapIV contains 25 RILs, which has six same SNPs to DN422, and two same SNPs to KY131. The mean PSST of the 44 HapI and 29 HapII RILs was 0.63 and 0.91, respectively, indicating that RILs with HapII of KY131 genotype usually have a higher mean PSST. Haplotype analysis demonstrate a key role of *LOC*_*Os06g39750* in regulating the PSST of rice once again.

## Discussion

Cold stress is one of the major abiotic environmental stresses that significantly affects rice yield. Improving the cold tolerance of rice varieties using cold-tolerant genes is a fast and efficient approach to reduce the yield loss in rice cultivation regions. KY131, a *japonica* rice, has been widely cultivated in the northeast region of China for nearly 20 years. A prominent characteristic of KY131 is the strong cold tolerance to cope with the frequent low temperatures that occur during the booting stage in this high-latitude region (Yao et al. 2012; Wang et al. 2016). In this study, a RIL population derived from a cross between DN422, a cold sensitive *japonica* rice, and KY131 was exposed in a cold-water environment over 3 years to acquire the PSST to map cold-tolerant genes. The identification of cold tolerance at the booting stage is more difficult compared to other agronomic traits in rice. Therefore, we divided the RILs into early maturing, middle-maturing and late-maturing groups, and irrigated them independently to evaluate the PSST of the parents and each RIL line. The critical temperature for the cold stress treatment at the booting stage of rice is 17–20 °C (Zhou et al. 2010). The cold-water temperature of 17 °C selected in this study could differentiate the PSST of the two parents significantly, which made the RILs vary widely (Table 1, Fig. 1). All the measures adopted in this study were effective at obtaining accurate PSST phenotypic data.

Conventional methods of fine mapping and map-based cloning of QTLs were very difficult for the development of high-density genetic map and a series of near-isogenic lines, especially the genetic population derived from *japonica* × *japonica* or *indica* × *indica* crosses. For example, a long time was spent from the preliminary mapping to the cloning of the cold-tolerance gene *Ctb1* (Saito et al. 1995; Saito et al. 2001; Saito et al. 2010). Seq-BSA technology provides a powerful, time-saving method to narrow the chromosome interval harboring the target genes/QTLs. Using the strategy of traditional QTL mapping combined with Seq-BSA, Zheng et al. (2016) rapidly mapped and identified a novel broad-spectrum resistance gene to rice blast (*Pi65(t)*). The *Pi65(t)* region has been narrowed to 60 Kb, which contains 4 predicted *R* genes. In the present study, a major QTL *qPSST6* that explained 30.45% of the phenotypic variation was initially mapped using the traditional QTL mapping method. Despite the relatively low density of the genetic map containing only 158 SSR markers, the 3 years of phenotypic data of PSST demonstrate a major QTL existing within the 28.4 cM interval between SSR markers RM20261-RM20356. By employing the Seq-BSA method, *qPSST6* was quickly delimited to a 1.81 Mb physical interval on chromosome 6 (Fig. 4), and two genes, *LOC_Os06g39740* and *LOC_Os06g39750*, were locked according to their annotated biological function as “responds to cold”. Thus, our study provided a fast and cost-effective strategy to identify the quantitative locus of complex trait variation. Some QTLs about cold tolerance at the booting stage have been reported in recent years. These QTLs were mainly distributed on chromosome 3 (Andaya and Mackill 2003b; Suh et al. 2010; Shirasawa et al. 2012; Zhu et al. 2015; Ulziibat et al. 2016), chromosome 4 (Saito et al. 2004; Saito et al. 2001; Xu et al. 2008; Endo et al. 2016; Zhang et al. 2017), and chromosome 7 (Suh et al. 2010; Zhou et al. 2010). Andaya and Mackill (2003b) and Oh et al. (2004) identified a QTL controlling spikelet fertility (*qCTB6*) and the days to heading (*dth6*), respectively, located on chromosome 6, but the two QTLs were approximately 16.4 Mb and 11.9 Mb distant to *qPSST6* in this study. Beyond those, we did not find any QTL loci distributed on chromosome 6. Thus, *qPSST6* was a new locus for cold tolerance at the booting stage in rice.

Among the 269 predicted genes identified by QTL mapping and Seq-BSA, *LOC_Os06g39740* and *LOC_Os06g39750* were taken into full consideration as the candidate genes of *qPSST6* according to the results of the GO annotation generated by Seq-BSA. The results of the qRT-PCR showed that *LOC_Os06g39750* was induced strongly by cold stress in the cold-tolerant parent KY131. Haplotype analysis also demonstrated the RILs with HapII of KY131 genotype usually have a higher mean PSST. These all suggesting that *LOC_Os06g39750* was the candidate gene of *qPSST6*. So far, there has not been any report about *LOC_Os06g39750* in rice. By searching the Arabidopsis Information Resource (TAIR, http://www.arabidopsis.org/) database using the gene sequence of *LOC_Os06g39750*, ten Arabidopsis homologous genes were identified (Additional file 2: Table S12). Eight of the ten genes were the members of the 3-ketoacyl-CoA synthase family involved in the biosynthesis of VLCFA (very long chain fatty acids). Among the ten Arabidopsis homologous genes, *At5g43760*, *At2g26640*, *At2g16280*, *At1g25450*, *At1g01120*, and *At2g26250* were annotated as functions of “response to cold” in TAIR, indicating that they may participate the regulation of reactions to cold in Arabidopsis. An expression profiling analysis revealed that most of the 3-ketoacyl-CoA synthase (KCS) family genes in Arabidopsis, including the above six genes, responded to cold stress (Joubes et al. 2008). Therefore, it is reasonable to predict that *LOC_Os06g39750* is the candidate gene for *qPSST6*. However, gene clone, genetic transformation, and further studies are needed to functionally validate this conclusion.

## Conclusions

The present study using the Dongnong422/Kongyu131 RIL population and a genetic linkage map containing 158 SSR markers identified a novel major QTL *qPSST6* for cold tolerance at the booting stage of rice in the 28.4 cM region on chromosome 6. By using the combined Seq-BSA strategy, *qPSST6* was narrowed to a region of 1.81 Mb, which contains 269 predicted genes. According to the results of the gene ontology (GO) analysis, *LOC_Os06g39740* and *LOC_Os06g39750* were annotated as “response to cold”. *LOC_Os06g39750* exhibited strong up-regulated expressions at the 5, 7, 9, 11, and 13 day after the cold-water treatment in KY131. Haplotype analysis also demonstrate a key role of *LOC*_*Os06g39750* in regulating the PSST of rice, suggesting that it was the candidate gene of *qPSST6*. This study provided a fast and cost-effective strategy to identify cold-tolerant genes at the booting stage in rice.

## Additional files

Additional file 1: **Figure S1.** Distributions of coverage depth on chromosomes of the sequencing samples. X-axe indicates the 12 chromosomes of rice. Y-axe indicates the log2 value of coverage depth corresponding to chromosome location. (DOC 1130 kb)

Additional file 2: **Table S1.** The PSST of T-pool and S-pool. **Table S2.** Sequences of specific primers for PSST candidate genes. **Table S3.** Sequences of specific primers for SNP loci in LOC_Os06g39750. **Table S4.** Heading time, plant height, panicle number, and grain yield per plant of parents and RIL population in 2014, 2015 and 2016. **Table S5.** Coefficients of correlation (r) among PSST and PSSN in 2014, 2015 and 2016. **Table S6.** QTLs for PSSN of rice detected in 3 years. **Table S7.** Summary of Seq-BSA data for each sample. **Table S8.** Annotation information of predicted genes for PSST. **Table S9.** nsSNPs between DN422 and KY131 along with their S-pool and T-pool. **Table S10.** Information of SNP/InDel in LOC_Os06g39740 and LOC_Os06g39750. **Table S11.** Haplotype of LOC_Os06g39750 in RIL population and mean PSST of each RIL. **Table S12.** The Arabidopsis homologous genes of LOC_Os06g39750. (XLS 174 kb)

## Abbreviations

BSA: Bulked-segregant analysis
COG: Cluster of orthologous groups of proteins
ED: Euclidean distance
GO: Gene ontology
KEGG: Kyoto Encyclopedia of Genes and Genomes
MAS: Marker assisted selection
NGS: Next-generation sequencing
NR: Non-redundant protein
PSSN: Percent seed set under normal water irrigation
PSST: Percent seed set under cold water treatment
PVE: Phenotypic variation explained
QTL: Quantitative trait loci
RIL: Recombinant inbred line
S-pool: Sensitive pool
SSD: Single seed descent
T-pool: Tolerant pool
