# Supplementary material for: Identification of a cold-tolerant locus in rice (Oryza sativa L.) using bulked segregant analysis with a next-generation sequencing strategy
Source: Rice (N Y). 2018 Apr 18;11:24. doi: 10.1186/s12284-018-0218-1 (PMC5906412; doi:10.1186/s12284-018-0218-1)
Supplement: Supplementary file 1 — Figure S1. Distributions of coverage depth on chromosomes of the sequencing samples. X-axe indicates the 12 chromosomes of rice. Y-axe indicates the log2 value of coverage depth corresponding to chromosome location. (DOC 1130 kb) [file 12284_2018_218_MOESM1_ESM.doc]

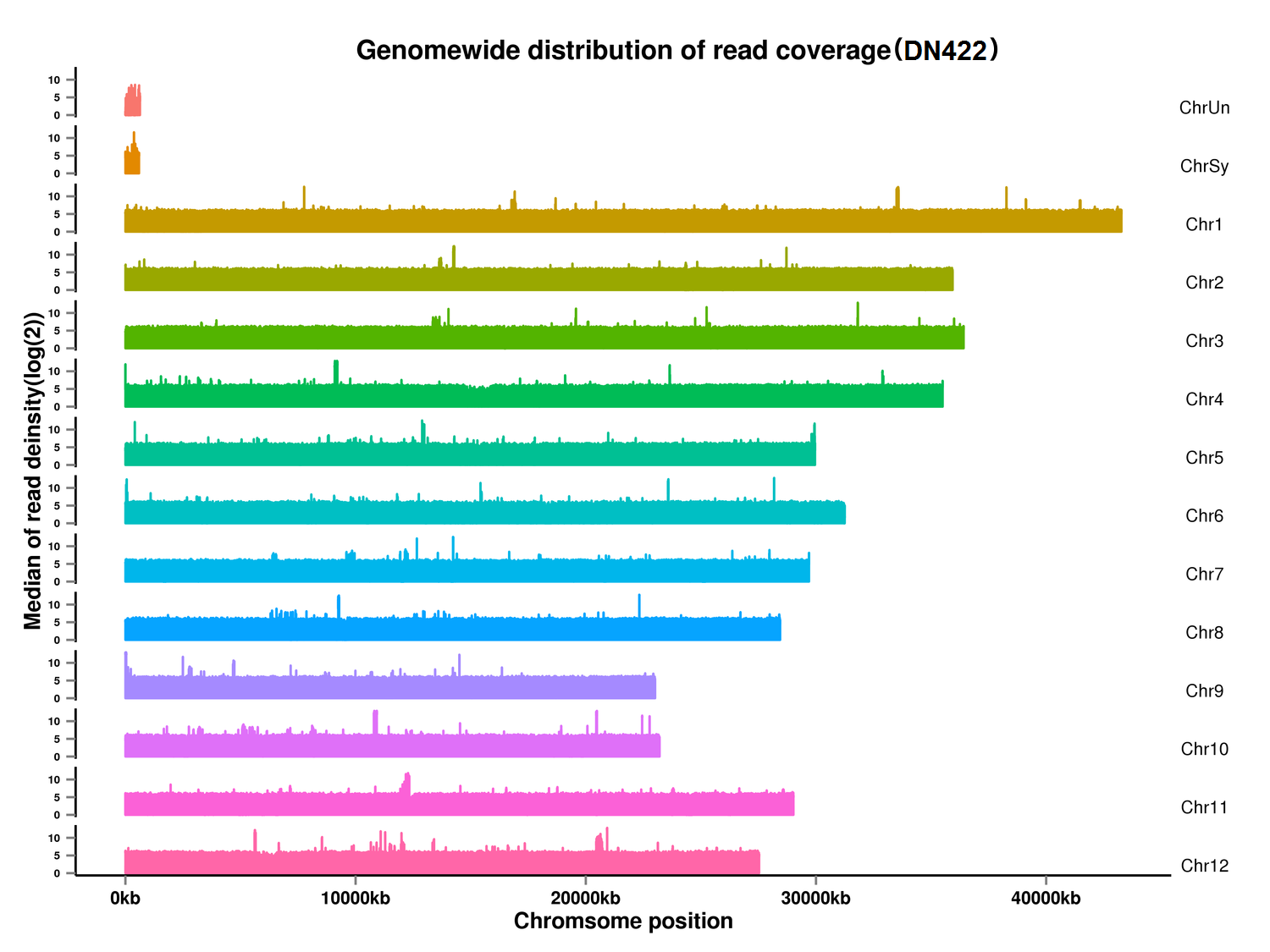


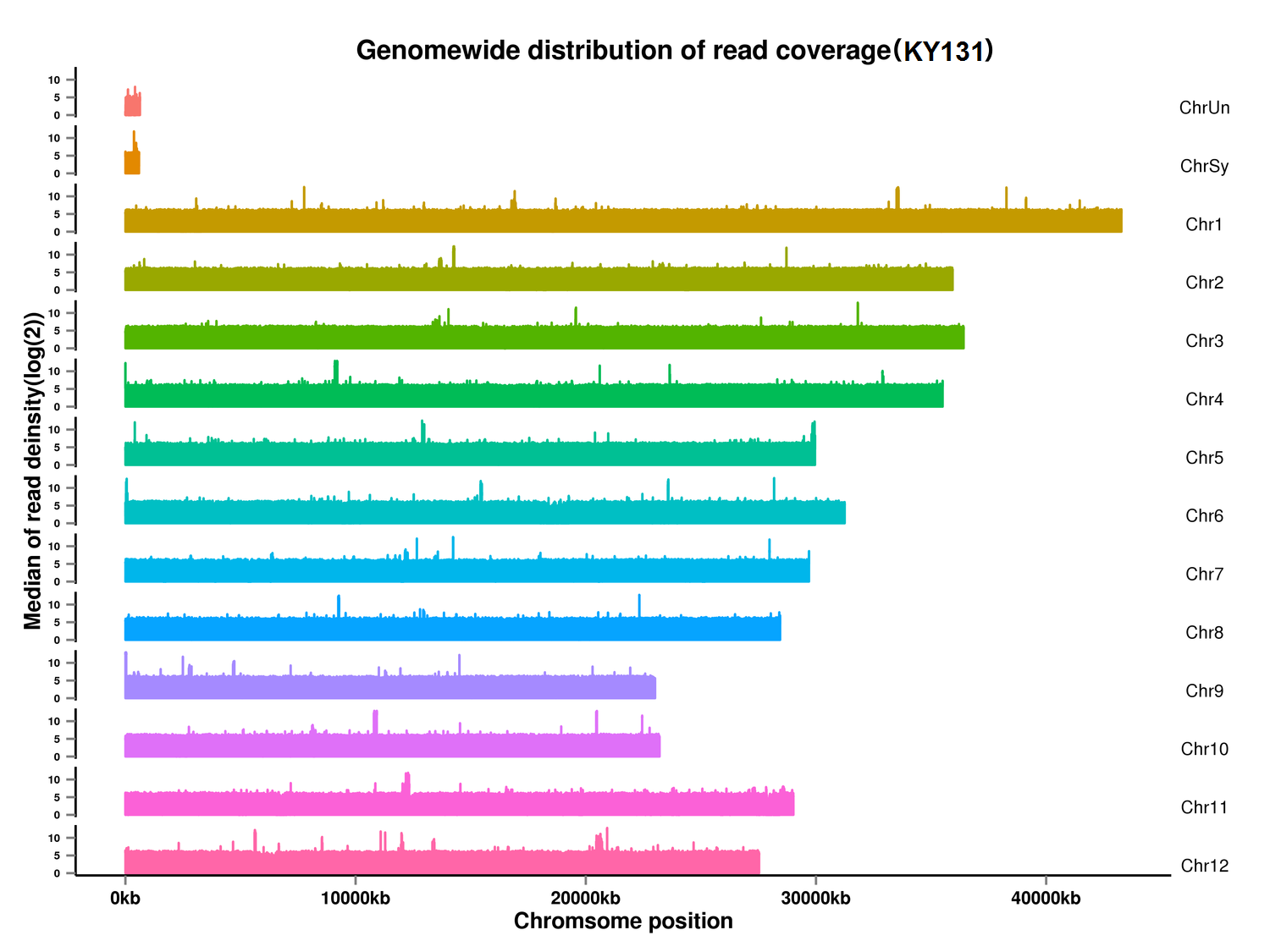


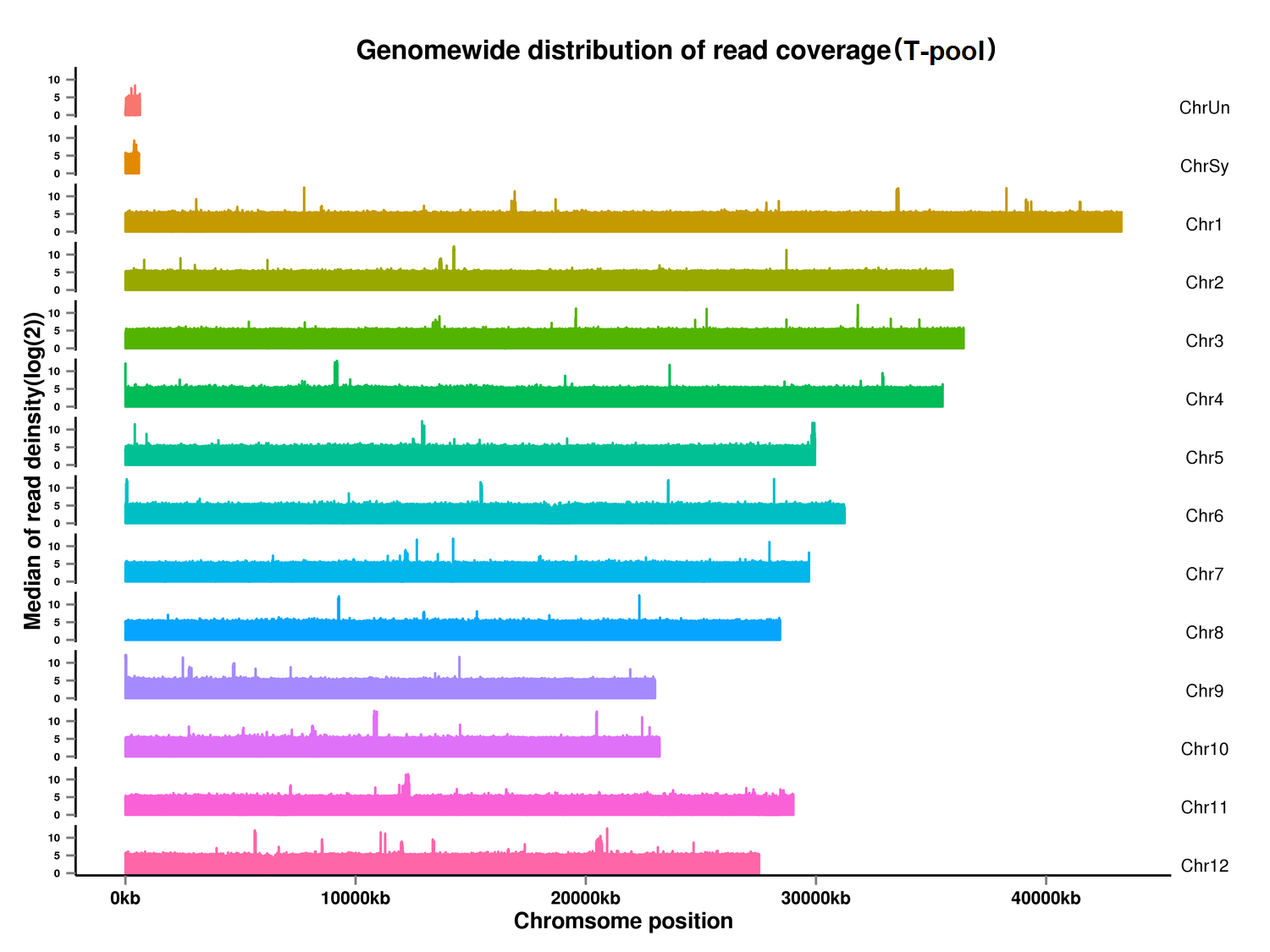


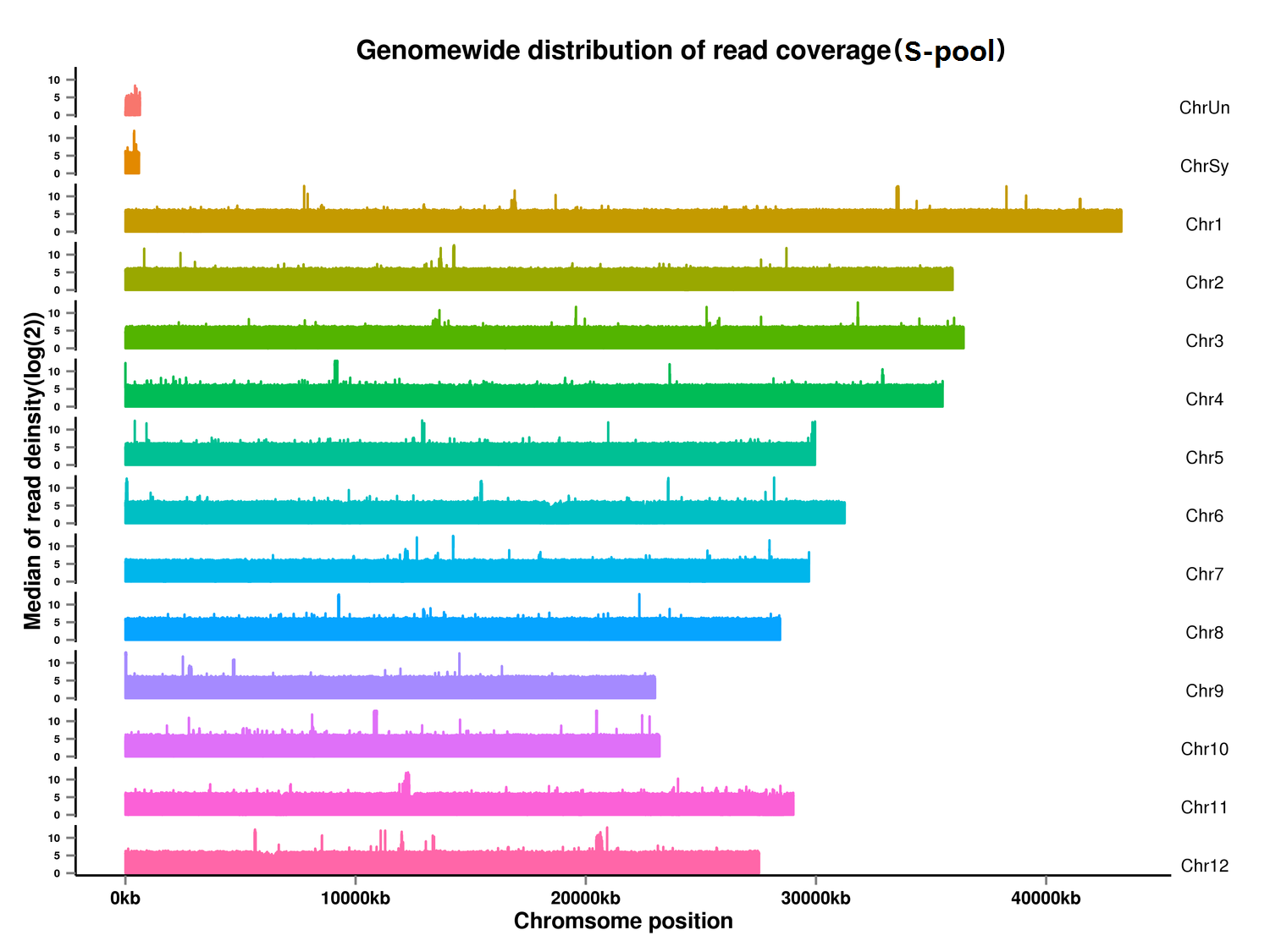


**Additional Figure S1**. Distributions of coverage depth on chromosomes of the sequencing samples. X-axe indicates the 12 chromosomes of rice. Y-axe indicates the log2 value of coverage depth corresponding to chromosome location.
